# Effects of litter quality on foraging behaviour and demographic parameters in *Folsomia candida* (Collembola)

**DOI:** 10.1002/ece3.10420

**Published:** 2023-08-18

**Authors:** Karolina Argote, Cecile H. Albert, Benoît Geslin, Charlotte Biryol, Mathieu Santonja

**Affiliations:** ^1^ Aix Marseille Université, CNRS, Université Avignon, IRD, IMBE Marseille France

**Keywords:** animal movement, Collembola, demographic parameters, foraging, litter quality

## Abstract

Litter quality has long been associated with demographic parameters of Collembola populations. However, little is known about the capacity of Collembola to perceive and seek better litter quality. To address this gap, three complementary laboratory experiments were carried out with the Collembola *Folsomia candida*. First, populations were fed on three different types of leaf litters (*Quercus pubescens*, *Acer opalus* and *Prunus avium*) and a control (agar‐agar‐brewer's yeast mixture) for 6 weeks to assess their impacts on demography (reproduction rate and population size). Second, the body length of individuals differentially fed with the same four types of resources was measured to assess a functional trait that can potentially affect movement parameters such as prospected area or foraging speed. Third, *F. candida* single individuals were exposed to the same litter quality gradient and placed at an increasing distance from the litter (from 1 to 5 cm). For 10 min, their foraging behaviour was recorded which included prospected area, foraging speed, perception distance and success in reaching the litter (foraging success). As expected, low‐quality litter (i.e. *Q. pubescens*) contributed to low population growth compared to the control treatment and the high‐quality litters (*P. avium* and *A. opalus*). In the third experiment, the probability of finding the resource was negatively correlated to the distance, but was unrelated to the litter quality and the Collembola body length. When resource was perceived, *F. candida* was able to switch from non‐directional to directional movements, with a large variability in the perception distance from a few millimetres to several centimetres. Taken together, our results indicate that litter quality plays a relevant role in Collembola demographic parameters once the population settles on litter patch, but not on foraging behaviour to select high‐quality resources.

## Graphical abstract

Embark on a captivating exploration of *Folsomia candida*, as we unravel the effects of litter quality on its foraging behavior and demographic dynamics. Discover how resource quality shapes carrying capacity and gain insights into unexpected foraging strategies.

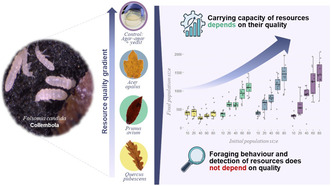

## 1 INTRODUCTION

Soil organisms play an essential role in ecosystem functioning due to their key implications in organic matter decomposition, nutrient availability for plant growth or soil structure maintenance (Nielsen et al., 2015; Santonja et al., 2017; Wardle et al., 2004). Among these, Collembola is a widespread and abundant group of soil microarthropods, occupying a wide range of trophic niches (Hopkin, 1997; Rusek, 1998). As microbivorous organisms (Hopkin, 1997), Collembola regulates microbial (bacterial and fungal) communities and their activities (Booth & Anderson, 1979; Filser, 2002; Song et al., 2020). They are also an important group of prey for generalist arthropod predators including spiders, centipedes and mites (Aupic‐Samain, Santonja, et al., 2021; Santonja et al., 2018; Vucic‐Pestic et al., 2010). As Collembola form a key component of the soil compartment and trophic networks, any changes in population dynamics and Collembola behaviour could have numerous impacts on soil food webs and associated ecosystem services (A'Bear et al., 2013; Santonja et al., 2017; Scheu, 2002).

Most species of Collembola are considered unspecialized feeders of fungal hyphae and spores, and bacteria, but also of non‐microbial sources, such as decaying plant debris, pollen, algae, decaying animals and faeces. Depending on the quality of their habitat, the season and the vertical distribution of the species, they can use one or many of these resources (Saur & Ponge, 1988). Several studies have reported that a high resource quality, expressed through a narrow C/N ratio favours the demographic parameters of Collembola such as reproduction and egg laying (Aupic‐Samain et al., 2019; Booth & Anderson, 1979; Sadaka‐Laulan & Ponge, 2000). For instance, Booth and Anderson (1979) reported a greater rate of moulting and a higher fecundity of *Folsomia candida* with increased nitrogen concentration. Likewise, Lavy and Verhoef (1996) reported that the nitrogen content of the resource had a direct influence on the *Orchesella cincta* growth rate.

Food resources being a strong component of Collembola population dynamics, several studies have focused on the ability of Collembola to perceive and choose a resource, for example through Y‐tube experiments (Booth & Anderson, 1979; Chauvat et al., 2014; Halliday et al., 2019; Menta et al., 2019). However, little is known about the relationship between litter quality and the ability of Collembola to perceive and select a high‐quality litter prior to population establishment and development.

In this context, we conducted three complementary laboratory experiments to investigate the effects of litter quality on population size, reproduction rate and foraging behaviour (i.e. when individuals seek food) of the Collembola species *F. candida*. Considering that the chemical composition of the leaf litter indicates its quality as a resource for decomposer organisms (Strickland et al., 2009), we used four types of resources (three litter types and a control treatment composed of an agar‐agar‐brewer's yeast mixture) chosen to form a resource quality gradient based on their nutrient concentrations (N, P, K, Ca, Mg, Na; Santonja et al., 2019). First, we hypothesized that increasing the nutrient concentrations of resources would induce higher growth and fecundity which could lead to larger individuals and higher population size. However, before a population can develop on a suitable litter habitat, Collembola must detect the resource. Considering that *F. candida* is blind and although partly light‐sensitive (Auclerc et al., 2010; Gallardo Ruiz et al., 2017), this species must thus rely on olfactive cues or chance (random movement) to detect distant resources. The foregoing leads us to predict that in the presence of low‐quality resources, the prospected area could increase. If the Collembola are not directly attracted by the resource they should wander around more or less randomly which should increase the prospected area.

## 2 MATERIALS AND METHODS

### 2.1 Material collection

#### 2.1.1 Selection of a gradient of litter quality among different leaf‐litters

Leaves were collected on the Lure Mountain, located in the ‘*Les Monts de Vaucluse*’ mountain chain, in south‐eastern France (44°07′22.7″ N 5°48′09.2″ E). At this supra‐Mediterranean stage (between 800 and 1200 m a.s.l.), the forest is dominated *by Quercus pubescens Willd*. and two companion species (*Acer opalus Mill*. and *Prunus avium* L.). Senescent leaves were collected during litterfall in November–December 2019 using litter traps under a tree canopy. Prior to the laboratory experiment, to allow the leaching of secondary metabolites potentially toxic to Collembola (Asplund & Wardle, 2013; Chomel et al., 2014) and the colonization of microorganisms (Aupic‐Samain, Baldy, et al., 2021; Santonja et al., 2018), leaves were placed in litterbags and disposed on the soil surface at the Oak observatory at the Observatory of Haute‐Provence (O_3_HP experimental site; 43°56′6.72″ N 5°42′38.52″ E) near the Lure Mountain. After 40 days, litter samples were collected, dried at room temperature for 24 h and then frozen twice at −18°C for 48 h to remove soil fauna (Aupic‐Samain et al., 2019; Santonja et al., 2018). Samples of litter species were stored in a dark room at ambient temperature until the start of the experiment, except four aliquots of each litter which were ground into a powder prior to chemical analyses.

We selected these three species in order to create a gradient of leaf litter quality based on their nutrient concentrations (Table 1). Nitrogen (N) concentration was determined by thermal combustion in a Flash EA 1112 series C/N elemental analyser (Thermo Scientific). The phosphorus (P), potassium (K), magnesium (Mg), calcium (Ca) and sodium (Na) concentrations were measured by the Laboratoire Teyssier. Phosphorus concentration was measured colourimetrically using the Olsen method. K, Mg, Ca and Na concentrations were estimated using an atomic absorption spectrometer. To categorize the litter types (poor vs. high litter quality), a principal components analysis (PCA, *dudi.pca* function from *ade4* package) was performed using the scaled values of the six concentrations (four replicates per measurement; Figure S1). The differences in nutrient concentrations between litter species were assessed using one‐way ANOVAs (*anova* function from *car* package) followed by Tukey's tests (*glht* function from *multcomp* package) to carry out post hoc pairwise comparisons (Table 1). *Acer opalus* had a higher N content, while *P. avium* had higher P, K, Mg and Ca contents than the other litters (Table 1).

**TABLE 1:** Main initial leaf litter characteristics of the three litter types.

	*Quercus pubescens*	*Acer opalus*	*Prunus avium*	*F*‐ratio	*p*‐value
N (mg g^−1^ dw)	0.64 ± 0.01a	0.82 ± 0.01b	0.79 ± 0.01b	**39.78**	**<.0001**
P (mg g^−1^ dw)	0.79 ± 0.02b	0.52 ± 0.01a	0.80 ± 0.03b	**12.09**	**.0028**
K (mg g^−1^ dw)	1.11 ± 0.03a	3.51 ± 0.12b	5.34 ± 0.08c	**157.4**	**<.0001**
Mg (mg g^−1^ dw)	2.18 ± 0.02a	3.00 ± 0.10b	4.49 ± 0.09c	**59.09**	**<.0001**
Ca (mg g^−1^ dw)	17.78 ± 0.28a	27.41 ± 0.34b	30.87 ± 0.19c	**133.1**	**<.0001**
Na (mg g^−1^ dw)	0.032 ± 0.001a	0.027 ± 0.001a	0.032 ± 0.001a	2.058	.1840

*Note*: Significant effects are indicated in bold. Values are mean ± SE, *n* = 4. Separated one‐way ANOVAs were performed to test the effects of litter type on initial litter characteristics. Different letters denote significant differences among litter types with a < b < c.

Abbreviations: Ca, calcium; K, potassium; Mg, magnesium; N, nitrogen; Na, Sodium; P, phosphorus.

#### 2.1.2 Preparation of Collembola cohorts

*Folsomia candida* (Willem 1902) is the most intensively studied Collembola species (Fountain & Hopkin, 2005). As a parthenogenetic ubiquitous species with a short reproductive cycle, *F. candida* is easy to maintain and study under laboratory conditions (Usher & Stoneman, 1977).

**FIGURE 1:**
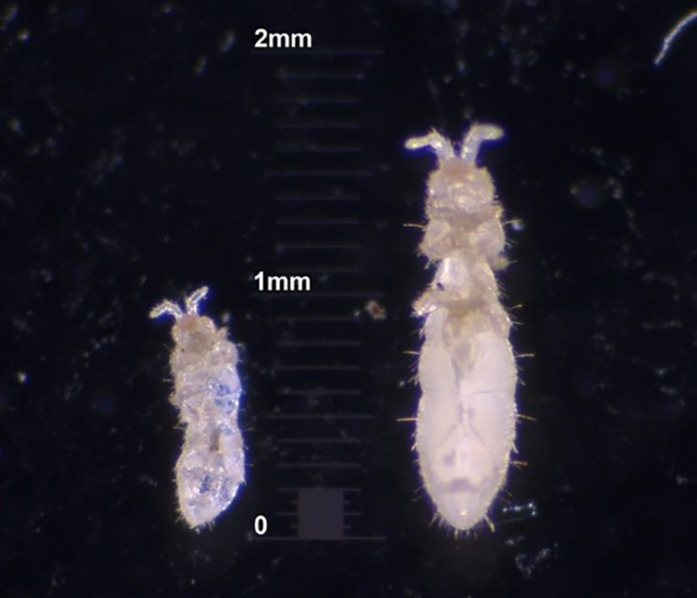
Pictures of *F. candida*: a juvenile individual on the left (1.0 mm in length and 10 days old) and a sub‐adult individual on the right (1.7 mm in length and 19 days old). ©Photo by Karolina Argote.

The population used in the present study was obtained from the rearing of the Mediterranean Institute of Biodiversity and Ecology (IMBE; Figure 1). Individuals were reared in plastic boxes (5.5 cm diameter × 9.5 cm high) containing a flat mixture of plaster of Paris and activated charcoal in a ratio of 9:1. Boxes were kept moist in a room at 20 ± 2°C. To use similar cohorts for the experiment, adults were placed in refrigerators at 4°C for 2 days to induce a thermic stress and stimulate egg‐laying. After oviposition, adults were removed and the eggs hatched 3–4 days later. To ensure that the population was as homogeneous as possible, eggs were placed in a large container and juveniles were fed for the first time altogether with dry yeast pellets. We used 19–22 days‐old individuals that underwent a 48‐h fasting period before the start of both experiments, in order to have more active individuals when exposed to a resource.

### 2.2 Experimental setup

#### 2.2.1 Experiment 1: Effect of litter quality on demographic parameters

The effect of litter quality on demographic parameters of *F. candida* populations was assessed in microcosms (plastic jars of 5.5 cm diameter × 9.5 cm height). We used the three selected litter types *Q. pubescens*, *A. opalus* and *P. avium* and a control treatment composed of an agar‐agar and dried brewer's yeast mixture (ratio of 2:1), as dry brewer's yeast is known to be an appreciated food for this Collembola species which enables rapid growth and high fecundity, and thus provides a positive control treatment under optimal rearing conditions.

The four selected types of resources were crossed with five initial population sizes (10, 20, 40, 60 or 80 individuals). Individuals (19–22 days old) were introduced into microcosms previously filled with the same plaster and charcoal substrate as the rearing boxes (see above). Each combination was replicated 12 times, which led to a total of 240 microcosms ([3 litters + control] × 5 initial population sizes × 12 replicates).

**FIGURE 2:**
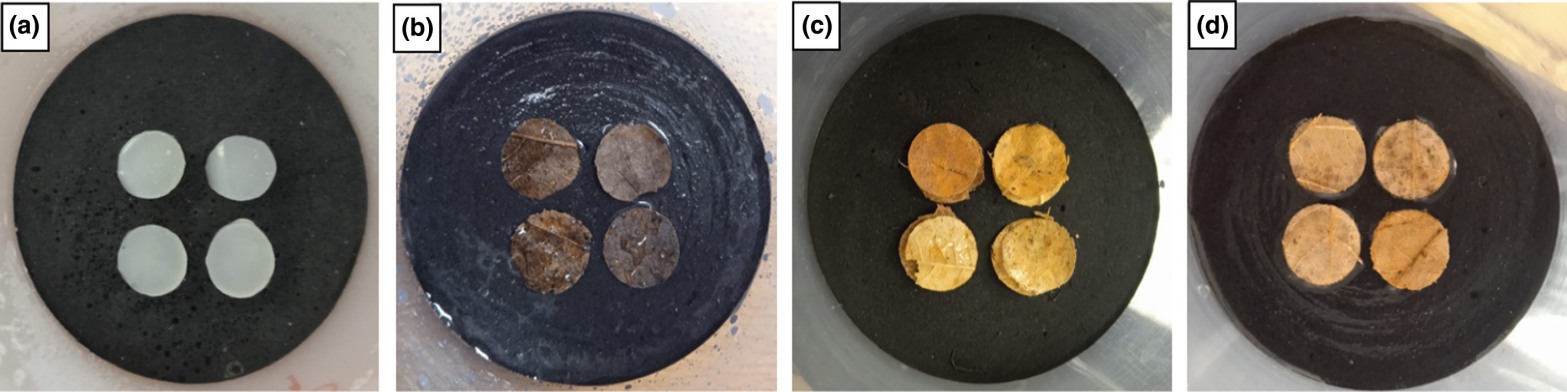
Microcosms with four different types of resources for the density‐dependence experiment: (a) agar‐agar‐brewer's yeast mixture (4 discs), (b) *Prunus avium* (4 discs), (c) *Acer opalus* (8 discs), (d) *Quercus pubescens* (4 discs). For the *Acer opalus* species, the number of discs was doubled as each disc's dry weight was 50% lower than the dry weight of the discs of the other litter types.

Two weeks before the experiment, four 1‐cm diameter discs of leaf litter were added to the microcosms in a square spatial configuration (Figure 2) with two drops of a 1:1 solution of distilled water and brewer's yeast per disc to stimulate microbial growth (Hopkin, 1997). To use the same amount of litter for all three tree species, the *A. opalus* discs were doubled (i.e. two discs sealed on top of each other due to humidity) since the weight of a disc of *Q. pubescens* or *P. avium* leaves is twice the dried weight of a disc of *A. opalus* leaves.

The experiment began with the addition of individuals according to the above‐mentioned protocol. Microcosms were kept in a climate‐controlled room at 20 ± 2°C under natural photoperiod for 6 weeks. Once a week they were ventilated and hydrated using distilled water to maintain constant humidity. At the end of the experiment, each microcosm was flooded with 70% ethanol, to collect all individuals and store them in clean jars for later counting. Count was performed using a binocular stereo‐zoom microscope (*Carl Zeiss model Stemi 305*) to estimate population size and reproduction rate. The population size was determined as the total number of individuals per jar at week six. The reproduction rate was calculated as the ratio between final and initial population sizes.

#### 2.2.2 Experiment 2: Effect of litter type on body length

We expected individuals raised on nutrient‐rich resources to grow faster than those raised on nutrient‐poor resources. To explore this hypothesis, we measured the body length of individuals (i.e. total length in mm), using a digital microscope (*Zeiss Axiocam 105* microscope camera, colour, CMOS, 1/2.5″, 5 megapixels).

Sets of individuals were fed from birth using the same quality gradient as in experiment 1 (three litter types and a control treatment with agar‐agar and dried brewer's yeast mixture). During the one‐month rearing, we regularly removed the eggs to maintain a constant density without juveniles. Eighty individuals were randomly selected for body length measurement (20 per litter type and 20 for the control treatment). To obtain the total length of the individuals we added two measurements: head (without antennae) and the thorax with the abdomen (without furca).

#### 2.2.3 Experiment 3: Effects of litter type and release distance on *F. candida* foraging behaviour

We then tested the effect of the same litter types on the foraging behaviour and the capacity of detection of Collembola. To do so, we crossed the litter quality gradient with a distance gradient, with individuals released at increasing distances from the litter discs (1, 2, 3, 4 and 5 cm from the resource). Each litter quality × distance combination was replicated 12 times, leading to a total of 240 runs ([3 litter qualities + control] × 5 initial distance × 12 replicates).

Runs were performed in an artificial squared arena consisting of 50 × 50 × 10 cm plastic boxes filled with the same plaster and charcoal substrate as in rearing boxes, providing a dark background to facilitate observation of the individuals. For each run, we added a 1‐cm litter disc at the centre of the arena. Disks were immersed for 3 days before the experiment in a solution of 100 mL water and 2 g of brewer's yeast to stimulate microbial growth.

**FIGURE 3:**
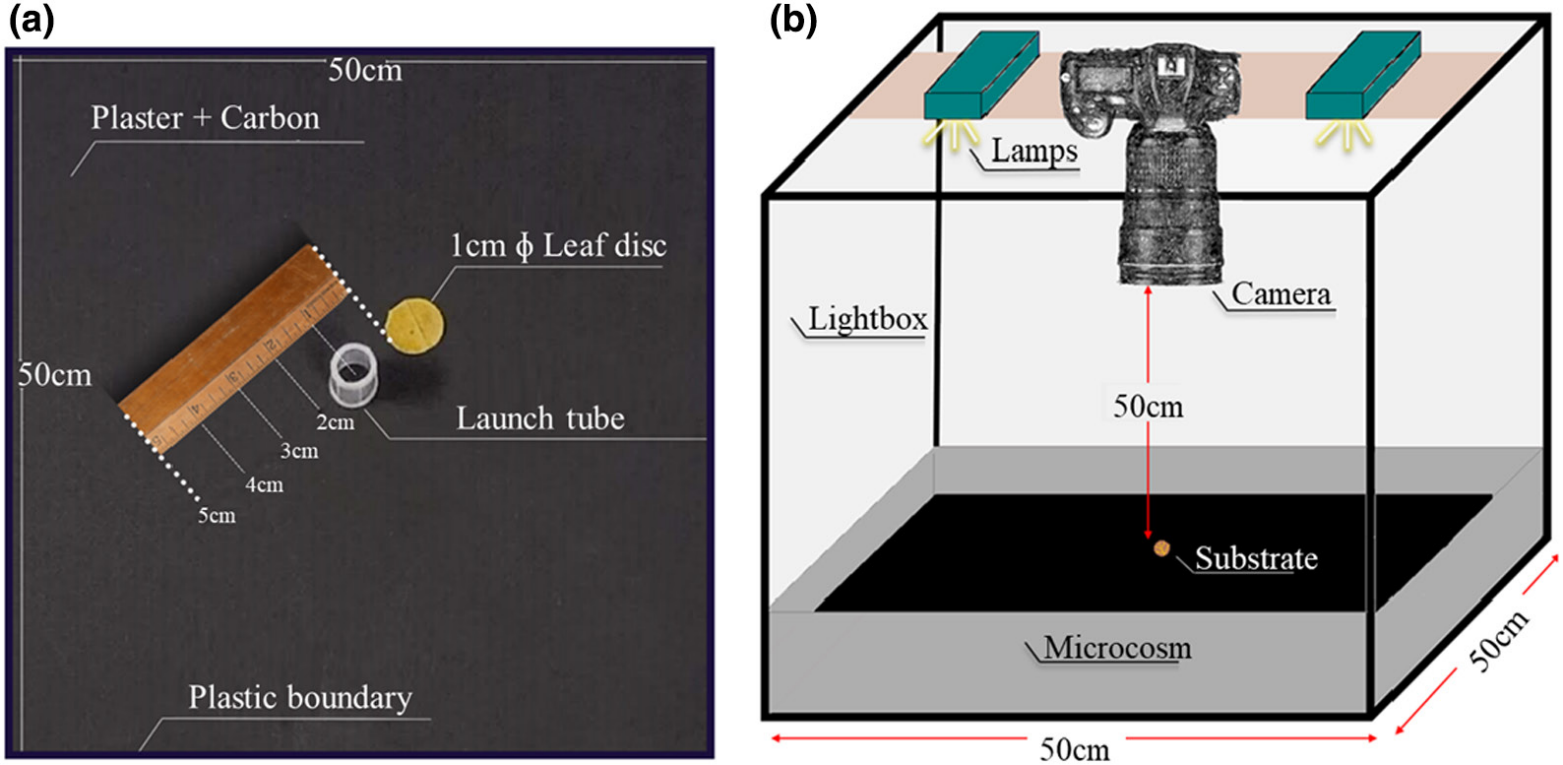
Schematic design of the laboratory set‐up for the detection Experiment 3. (a) 50 × 50 cm microcosm composed of a plaster of Paris + activated charcoal medium (ratio 9:1), 1‐cm diameter leaf disc and a hollow tube for the pre‐acclimatization of the Collembola. (b) Experimental device used to record the movement of the Collembola.

Individuals were released at a specific distance from the disc after a 2‐min acclimation period in a hollow transparent tube (0.5 cm in diameter × 2 cm in height; Figure 3a). Each run began after the acclimation period during which the individual is allowed to move freely in the arena for 10 min. Pictures of the arena were taken every 2 s with a *Nikon NIKKOR Z 24‐70 mm f/2.8 S* camera (Figure 3b), giving a total of 300 photographs per run. The arenas were cleaned with ethanol after each run to remove pheromones or any other chemical residues that might influence animals' movements.

For each run, we extracted the position (*x*, *y*) of the individuals over time (one position per photograph, that is, 300 positions per individual) following Mallard et al. (2013). Five movement parameters were recorded:

1. *The foraging success* defined as the success of an individual in reaching the litter disc. We considered a success when the animal approached the disc at a distance equal to or less than the length of its body length. The first point where the individual touches the litter disc was manually assessed for each successful run (Figure 3a).
2. *The foraging success time* (in s) defined as the time taken by individuals to reach the litter disc. To analyse this parameter, we did a *time‐to‐event analysis*. Based on the foraging success dataset in the function of the release distance, we analysed the expected duration of time until the individual reaches the resource.
3. *The perception distance* (in cm) associated with a foraging success: corresponds to the distance from the disc at which the individual switches from a non‐directional movement (exploratory foraging) to a directional movement towards the resource in successful cases (Auclerc et al., 2010). To do so, we analyzed the pattern formed by the distance traveled along the trajectory as a function of the Euclidean distance to the litter disc as recommended in Auclerc et al. (2010) (Figure 3b). This relationship converges to a straight line along the bisector as individuals move towards resources. We identified the point that corresponds to a change in the movement pattern—from random to directional movement—as the one corresponding to the last sharp angle along this relationship between the two distances (Figure S2, for examples).
4. *The foraging speed* (in cm s^−1^) is the average movement speed calculated as the mean distance travelled per time step in cm and divided by the 2 s‐time intervals (all individuals). It reflects a level of foraging activity of the individual and could be stimulated by an attractive litter.
5. *The prospected area* (in cm^−2^) is the total area explored by the individual during foraging (all individuals). We cut the picture into a grid of 150 × 150 pixels and we counted the number of grid cells visited at least once during the 10‐min foraging (Figure S3). After testing different aggregation levels from 20 × 20 to 600 × 600 pixels per grid cell, we chose 150 × 150 pixels per grid cell because it allowed a good ranking of the individuals (high variability of the values with an almost normal distribution). This area reflects the propensity of the individual to explore the arena.

**FIGURE 4:**
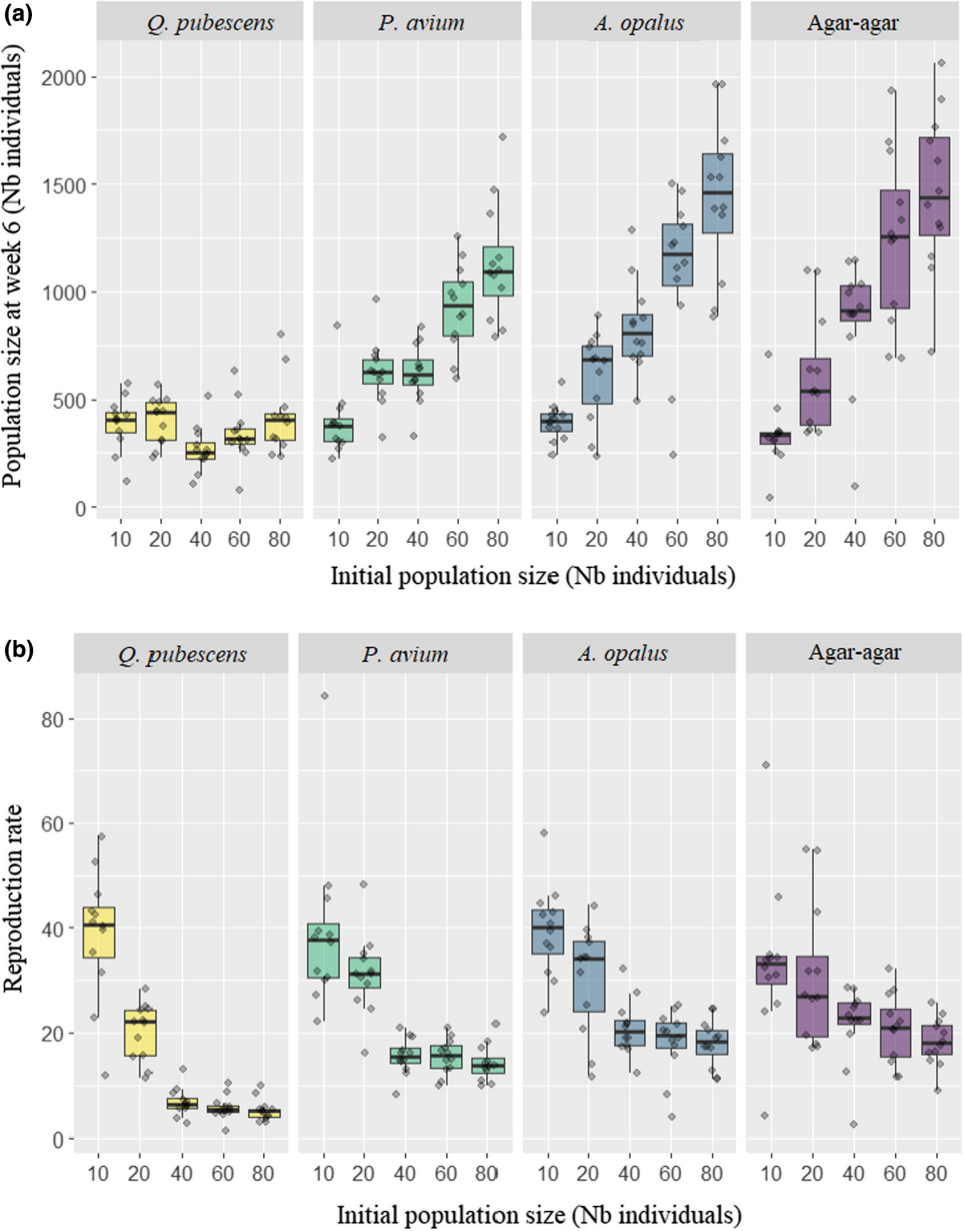
Population size (a) and reproduction rate at week six (b) according to the type of resource and the initial population size. The boxes are drawn from Q1 to Q3 with a horizontal black line denoting the median value.

A different *F. candida* individual was used for each test. As mentioned above for the experiment 2, all individuals used in this third experiment were fed from birth with the same resource they encountered during the run. The experiment was carried out using two 90‐lumen LED lights each, arranged at a height of 50 cm (Figure 4b). A different square arena was used for each test, previously cleaned using a 40% ethanol dilution. Once the ethanol has evaporated, the arena was hydrated 20 min before each test, always using the same amount of water (100 mL distributed evenly throughout the landscape), in order to have a comparable humidity between tests.

### 2.3 Statistical analyses

For Experiment 1, to test the effect of resource type and initial population size on the population size at week six, we used a generalized linear model (hereafter GLM) with a quasi‐Poisson error distribution and log link function. We compared the potential models (resource type or population size, both and their interaction as explanatory variables) based on their Akaike Information Criterion (hereafter AIC). Similarly, we used a GLM with a negative binomial error distribution and log link function to test the effect of resource type and initial population size on the reproduction rate. To determine the effects of the different resource types on the population size at week six and on the reproduction rate, we applied a post hoc multiple comparison test using the Holm correction method to adjust *p*‐values.

For Experiment 2, in order to test the effect of the resource on the individual body length (*N* = 80) we used a Kruskal–Wallis test because the assumptions of normality of the residuals were not met with parametric analysis. Next, we applied Dunn's post hoc multiple comparison test, to determine differences between groups, using the Holm correction method to adjust *p*‐values. Likewise, we tested individual body length effects on movement parameters. For this, we applied a GLM with a binomial error distribution and logit link function for foraging success, a normal error distribution and log link function for prospected area and foraging speed, and an inverse Gaussian error distribution and log link function for perception distance.

For Experiment 3, to test the effect of resource type and release distance on foraging behaviour, we used GLMs with a binomial error distribution and logit link function for foraging success, a normal error distribution and log link function for prospected area and foraging speed and finally a gamma error distribution and log link function for perception distance. We compared potential models (resource type or release distance, both and their interaction as explanatory variables) based on their AIC. Next, we applied a time‐to‐event analysis (also known as survival analysis) using the nonparametric Kaplan and Meier (1958) estimator for the success in foraging and the time of its achievement in function of the release distance and the log‐rank test (also known Mantel‐Cox test) to compare differences between groups. Then we plotted the time‐to‐event function.

All statistical analyses were performed using R software (version 4.1.3; R Core Team, 2022) and all the graphics were produced using the package ggplot2 (Wickham, 2016). Modelling assumptions such as normality and homoscedasticity of residuals were checked using the Shapiro–Wilk and Levene tests as well as the package DHARMa v0.4.6 (Hartig & Lohse, 2022). Additionally, other packages for R were used, such as: *multcomp* v1.4‐16 (Hothorn et al., 2008) as well as *FSA* v.0.9.3 (Ogle, 2018) for multiple comparisons analysis, *ade4* v1.7‐20 for litter quality principal components analysis (PCA; Dray & Dufour, 2007), *MASS* v7.3‐58.1package (Venables & Ripley, 2013) for negative binomial GLM, and *AICcmodavg* v2.3‐1 package for AIC model comparisons (Mazerolle, 2020).

## 3 RESULTS

### 3.1 Relationship between resource quality and Collembola demographic parameters

The best model to explain the population size after 6 weeks included resource type, initial population size and their interaction. The final population size (week 6) increased with the initial population size for the three higher quality resources (agar‐agar, *A. opalus* and *P. avium*), while it remained constant with *Q. pubescens* (Figure 4a). At a small initial population size (10 individuals), the final populations were similar for the different resources (Figure 4a, Table S1). For a larger initial population size (80 individuals), populations reared on *Q. pubescens* were significantly smaller compared to the other three resources (Figure 4a, Table S1). For intermediate cases (20, 40 and 60 individuals) resources formed a gradient with agar‐agar‐brewer's yeast mixture leading to the biggest population, *Q. pubescens* to the smallest population and the other two litters to intermediate population size at week six (Figure 4a, Table S1). While *A. opalus* and *P. avium* led to similar population sizes for 10, 20 and 60 individuals in the initial population, *A. opalus* also led to larger populations than *P. avium* and equivalent to agar‐agar for 40 and 80 individuals in the initial population (Figure 4a, Table S1).

Likewise, the best model to explain the reproduction rate included resource type, initial population size and their interaction. The average number of individuals produced per Collembola during the six‐week period was 38, 28, 16, 15 and 14 for the initial population sizes of 10, 20, 40, 60 and 80 individuals, respectively. Again, when the initial population size was small (10 and 20 individuals), resource type had no effect on the reproduction rate (Figure 4b). When the initial population size was larger, *Q. pubescens* led to a lower reproduction rate (Figure 4b, Table S2). *Prunus avium* and *A. opalus* marginally led to an intermediate reproduction rate while agar‐agar‐brewer's yeast mixture led to the highest one (Figure 4b, Table S2). For all resources, the reproduction rate declined with increasing initial population, mildly for agar‐agar‐brewer's yeast mixture and more drastically for *Q. pubescens* (Figure 4b, Table S2).

### 3.2 Effects of resource quality and distance on Collembola foraging behaviour

#### 3.2.1 Response of body length to resource quality and its effect on movement

**FIGURE 5:**
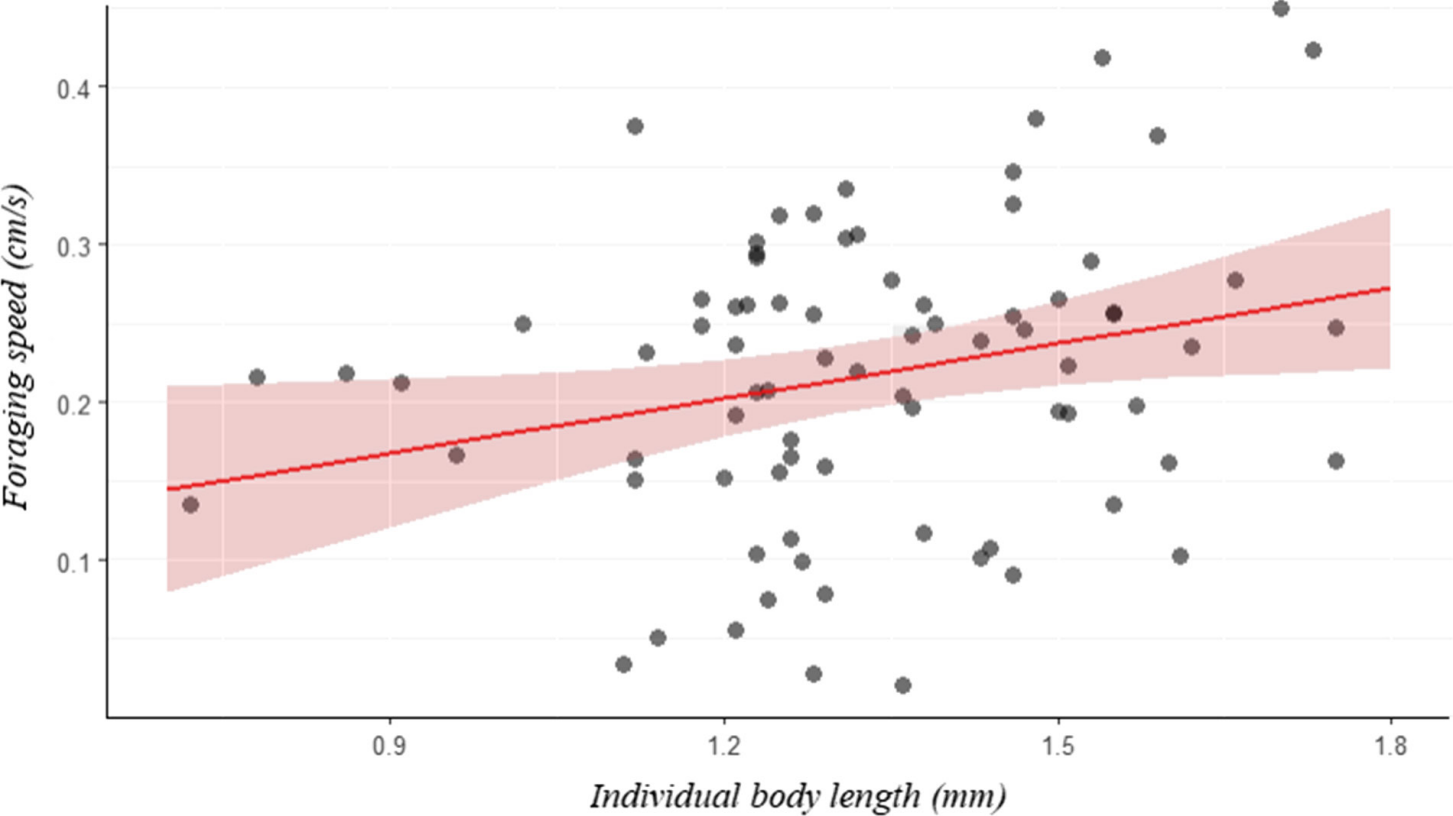
Marginal effects plot for the best‐fitted model between foraging speed and individual body length. The 95% confidence intervals are shown as shaded areas. *R* ^2^ = 0.93, *p* = .021, *N* = 80.

Contrary to our initial hypothesis, the resource quality on which the individuals were reared had no effect on the individual body length (*p* > .05). Regarding the mean foraging speed, although it was not affected by the type of resource, it increased significantly with the body length: each additional millimetre of body length increases the mean foraging speed by 0.58 mm s^−1^ (*p* = .021, *R* ^2^ = 0.93, Figure 5). However, no significant effects of the individual body length were found on other movement variables (prospected area, foraging success and perception distance).

#### 3.2.2 Foraging behaviour

**FIGURE 6:**
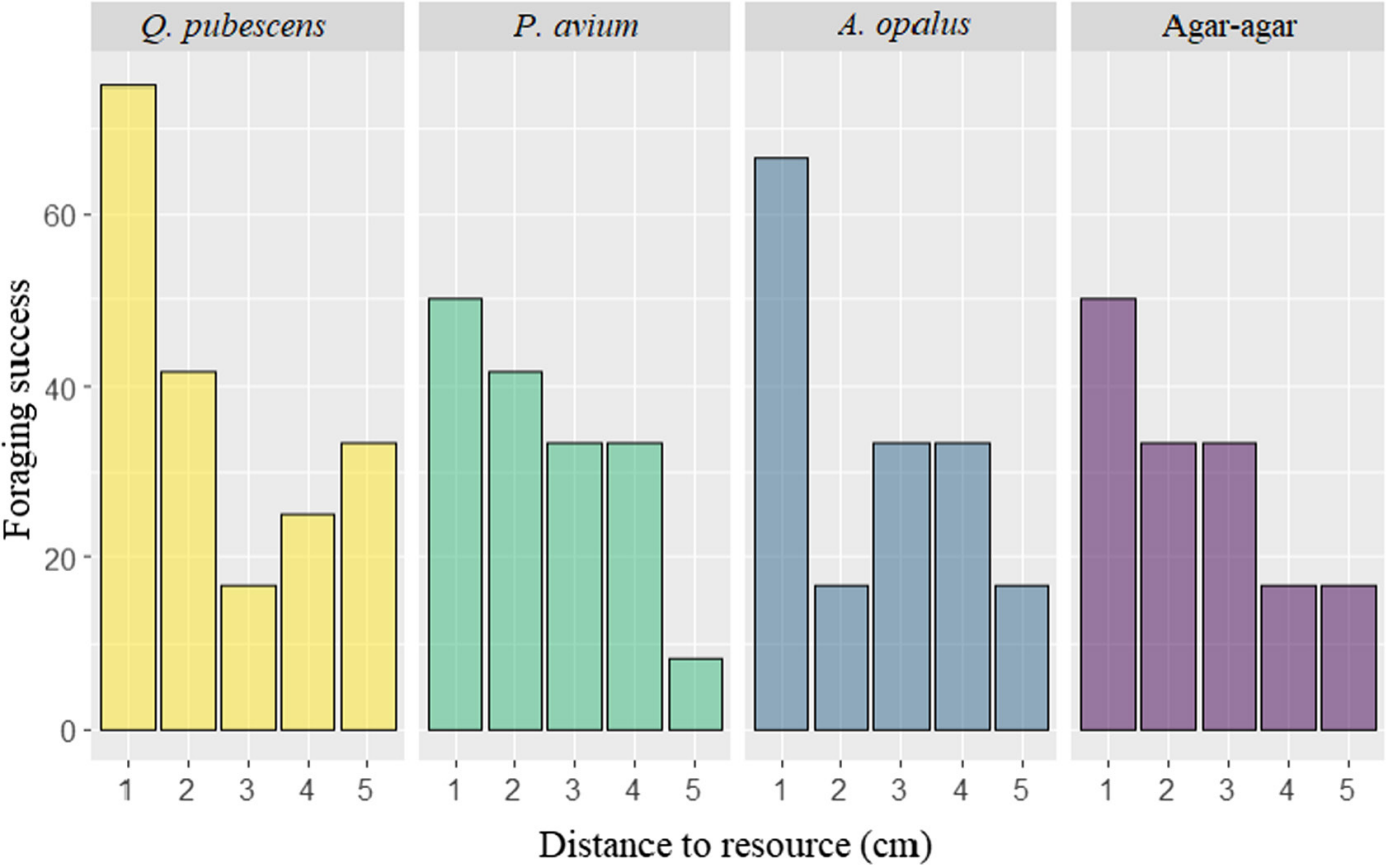
Probability of success in finding the resource according to the release distance to the resource. Each bar represents the frequency of success in finding resources for the 12 replicates per treatment (5 release distances × 4 types of resources).

**FIGURE 7:**
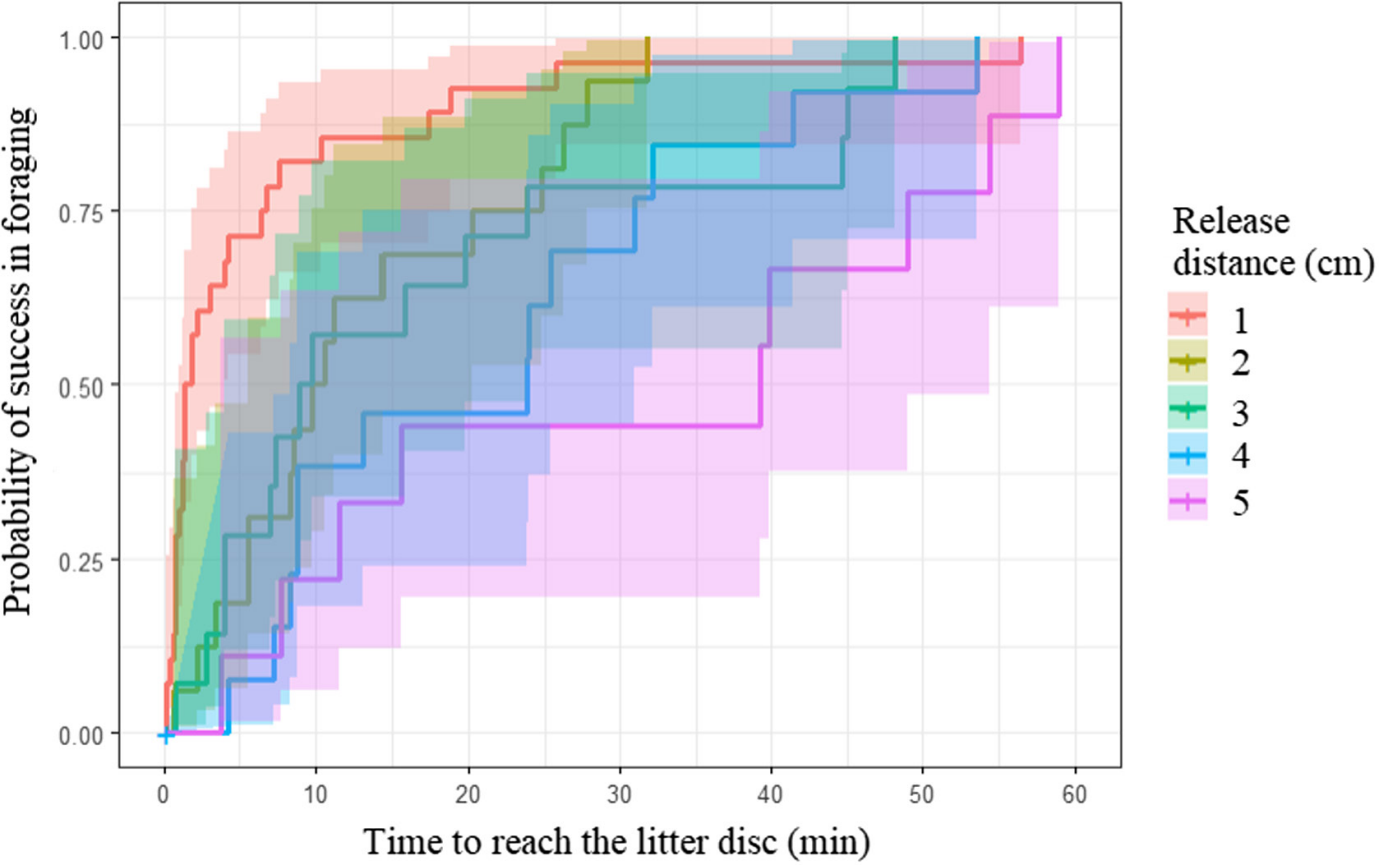
Kaplan–Meier plot of cumulative probability of success in foraging versus the time to reach the resource. The probability of success in foraging of the five groups is significantly different (*p* < .001). The shade around each line shows a 95% confidence interval.

Over the 240 runs, foraging success reached 34% (80 successful individuals). The best model to explain the foraging success of individuals included the release distance only. Resource quality had no effect on foraging success. Foraging success decreased non‐linearly with increasing release distance (*p* < .001, Figure 6), it drops drastically after 1 cm. Time‐to‐event analysis showed that at a time t, at short release distances, a higher percentage of individuals will have reached the litter disc than at longer distances. However, before 60 min, 100% of the individuals will have reached the litter disc for all distances tested (1, 2, 3, 4 and 5 cm; Figure 7, Table S3). After the first 5 min, 70% of the individuals have already reached the litter disc. This contrasts sharply with the 5 cm launch distance where, after 5 min, only 12% of the individuals had reached the litter. Regarding the foraging speed, we found no clear effect of release distance or resource type (best model with distance only is equivalent to the sum model, delta AIC = 0.49 and to the null model, delta AIC = 1.81). Similarly, we found no effect of release distance or resource type on prospected area (best model with distance only was equivalent to the null model, delta AIC = 0.24, to the sum model, delta AIC = 1.3 and to the resource only model, delta AIC = 1.5).

**FIGURE 8:**
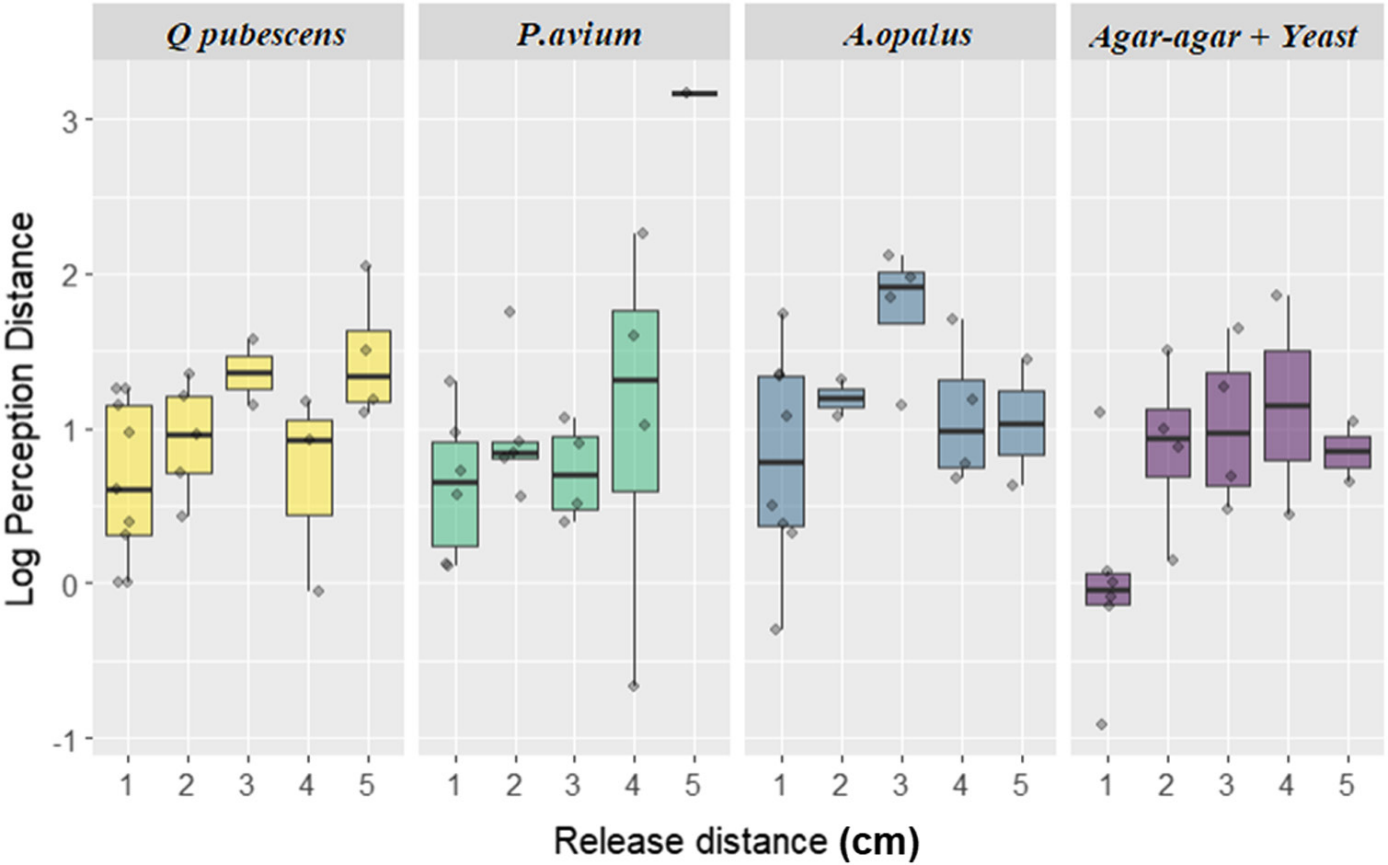
Perception distance in a logarithmic scale as a function of release distance by type of resource. The boxes are drawn from Q1 to Q3 with a horizontal black line denoting the median and the black dots represent the outliers.

Finally, we observed a large variability in the distance at which successful individuals changed their movement from random to directional (perception distance ranging from 0.40 to 23.84 cm, Figure 7). The best model to explain perception distance included release distance, resource type and their interaction (Figure 8). While agar‐agar, *P. avium* and *A. opalus* led to equivalent shorter perception distances, with no effect of release distance, *Q. pubescens* led to smaller perception distances that increased with release distance (*p* = .004).

## 4 DISCUSSION

Our complementary experiments demonstrate that the resource quality influences the population size and the reproductive rate of *F. candida*, but it does not influence body length, movement or detection of foraging individuals. Below we thus discuss the rationale and the implications of these different results.

### 4.1 Carrying capacity of resources depends on their quality

In our 6‐week experiment, the reared populations of *F. candida* were influenced by the combined effects of intrinsic (population density) and extrinsic (substrate quality) factors. Reproductive rates declined with initial population sizes, gradually for the high‐quality control resource and sharply for the three litter types. This suggests a density‐dependence effect where carrying capacity was reached between 20 and 40 individuals of initial population size. By considering the highest initial population densities, population sizes obtained after 6 weeks were larger with the agar‐agar resource—with which *F. candida* is normally reared in the laboratory to obtain its maximum reproductive potential—and smaller with *Q. pubescens* litter. At a high initial population density, Collembola may be highly constrained by the presence of congeners. For example, Mallard et al. (2020) reported that *F. candida* individuals grown in crowded conditions showed a reduced growth rate. Therefore, a high‐quality resource could support the coexistence of more individuals. The influence of litter quality on population dynamics is a fairly well‐documented subject for soil microarthropods. For example, Das and Joy (2009) observed that increasing litter quality (based on carbohydrate, sugar, protein and lipid concentrations) enhanced *Cyphoderus javanus* reproduction rate in a microcosm experiment, while Santonja et al. (2017) reported that increasing litter quality (based on nitrogen and phosphorus concentrations) in a Mediterranean oak forest was associated to higher Collembola abundance. Sánchez‐Galindo et al. (2021) also observed an increase in Collembola population size exposed to a high‐quality litter (*Cecropia andina*) in a litterbag experiment, while other studies performed in grassland demonstrated a positive effect of the presence of N‐rich legume species on Collembola population size (Birkhofer et al., 2011; Salamon et al., 2004).

### 4.2 Resource detection does not depend on their quality

Contrary to our initial hypothesis, the resource quality on which the individuals were reared had no effect on the individual body length of Collembola after 1 month of experiment. This finding contrasts with previous studies that reported a positive effect on resource quality on Collembola body length. For example, Sun et al. (2020) reported a positive effect of soil nitrogen addition on Collembola body length in the tropical Andes, while (Lavy & Verhoef, 1996) showed a positive relationship between *Orchesella cincta* body mass and the nitrogen content of hyphae of *Cladosporium cladosporioides* used as a resource.

Body length is very commonly correlated to movement speed in invertebrates, in general, larger body length is associated with reduced time costs because of higher achieved speed and increased perceptual range (Mech & Zollner, 2002). In our case, we observed no significant effect of the resource type on body size, but larger individuals tend to move faster (no significant effect on other movement parameters). Overall, we found no effect of resource quality either on the foraging success or on perception distance. Foraging success was only influenced by the distance at which individuals were released from the resource, declining abruptly beyond 1 cm. This suggests that the animal seeks‐finds something based on its proximity regardless of what type of resource it is, they rather find the disc randomly than because they are attracted by it. This is in line with Auclerc et al. (2010) that showed that Collembola can perceive a resource only a few cm away through olfactory cues (Salmon & Ponge, 2001). The fact that we found no effect of litter type on the detection rate could be potentially due to an absence of direct attraction by the volatile organic compounds released by the selected litters but by those released by the microbial communities colonizing these litters that were at a too early stage of development on the litter discs. Indeed, Moursi (1961) reported that Collembola sense and direct their movement towards CO_2_ sources associated with microbial activity, while Staaden et al. (2011) demonstrated that Collembola differentiates fungi using olfactory cues.

Interestingly, though the average perception distance—the distance at which we observed a change from random to the directional movement towards the resource—is rather small, we observed a high variability among successful individuals; while most individuals switched their behaviour at small distances, some individuals also switched after having explored quite large surfaces and being quite far from the resource (maximal observed distance = 24 cm). Success may therefore be more linked to chance at small distances and to a decision‐making process at large distances; because no other or better resource was found, the individual finally goes back towards a resource detected earlier during the foraging (Webb, 2019).

Beyond the detection distance, we also observed a high variability in foraging behaviour, despite individuals being clones. While looping movement is considered to be a sophisticated search strategy (Bengtsson et al., 2004), not all individuals used it and some moved rather in a straight way. Some also explored large distances, while others remained more confined. Although this is beyond the scope of our study, our results suggest that the variability in *F. candida* movement could be related to other factors such as their moult cycle, their age or their ‘micropersonality’ (Bailey et al., 2021; Palevody, 1974).

Finally, our results show that high‐quality resources that sustain large populations in *F. candida* do not particularly attract individuals more than low‐quality resources. As Collembola are highly sensitive to environmental conditions with potentially dramatic drops in populations when harsh winter or severe droughts occur, they have a capacity to (re)colonize resource quickly, independently of its quality. This quality rather plays a role later on population size and reproduction rate, but efficient/non‐selective inter‐population processes are key to maintain the species in the longer term (Hertzberg et al., 2000).

## AUTHOR CONTRIBUTIONS

**Karolina Argote:** Conceptualization (equal); data curation (equal); formal analysis (equal); methodology (equal); writing – original draft (equal). **Cecile H. Albert:** Conceptualization (equal); data curation (equal); formal analysis (equal); funding acquisition (equal); methodology (equal); project administration (equal); supervision (equal); writing – review and editing (equal). **Benoît Geslin:** Conceptualization (equal); data curation (equal); formal analysis (equal); methodology (equal); supervision (equal); writing – review and editing (equal). **Charlotte Biryol:** Data curation (equal); writing – review and editing (equal). **Mathieu Santonja:** Conceptualization (equal); data curation (equal); formal analysis (equal); methodology (equal); supervision (equal); writing – review and editing (equal).

## FUNDING INFORMATION

Our research was made possible by financial support from the ERC‐2020‐STG No. 949812. Funding agency played no role in the design of the study, the collection, analysis, and interpretation of data, nor in writing the manuscript.

## CONFLICT OF INTEREST STATEMENT

The authors declare that they have no known competing financial interests or personal relationships that could have appeared to influence the work reported in this paper.

## Supporting information


Data S1:

## Data Availability

The data supporting this article are available from the Dryad Digital Repository: https://doi.org/10.5061/dryad.rv15dv4dg.
