# Supplementary material for: Effects of litter quality on foraging behaviour and demographic parameters in Folsomia candida (Collembola)
Source: Ecol Evol. 2023 Aug 18;13(8):e10420. doi: 10.1002/ece3.10420 (PMC10439338; doi:10.1002/ece3.10420)
Supplement: Supplementary file 1 — Data S1: [file ECE3-13-e10420-s001.docx]

**Supporting information**

**Effects of litter quality on foraging behaviour and demographic parameters in *Folsomia candida* (Collembola)**

Karolina Argote*, Cécile H. Albert, Benoît Geslin, Charlotte Biryol, Mathieu Santonja

Aix Marseille Université, CNRS, Université Avignon, IRD, IMBE, Marseille, France

***Corresponding author:** Karolina Argote ([karolina.argote-deluque@imbe.fr](mailto:karolina.argote-deluque@imbe.fr))

**Supplementary Fig. S1.** Principal component analysis (PCA) biplot of chemical concentrations values (observations) of three litter types (*Acer opalus* (AO), *Prunus avium* (PA) and *Quercus pubescens* (QP)) on the variables (arrows): phosphorus (P), potassium (K), magnesium (Mg), calcium (Ca), nitrogen (N), and sodium (Na). The first two axes accounted for 84% of the variance. Observations that can be associated with a variable or variable group were delineated (dotted ellipses). The biplot shows a 95% confidence ellipse for a set of 2D normally distributed data samples.


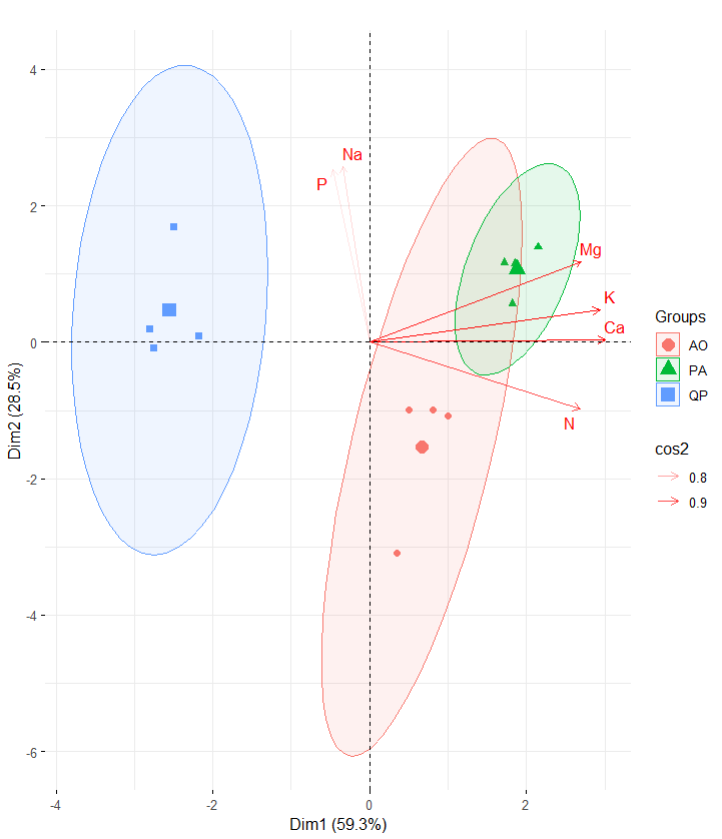


**Supplementary Fig. S2.** Three examples of different schemas of movement trajectories: A) Photo of examples in which the individual is successful in reaching the resource. Each red point represents the (x, y) position at time t (one position per photo) at which the individual switches from random to directional movement towards the resource. B) Traveling distance along trajectory *vs.* Euclidean distance for each position of the individual before it reaches the resource. The red point represents the (x, y) position when the individual changes its movement from a random to a directionally movement towards the resource. Blue points depict the release point.


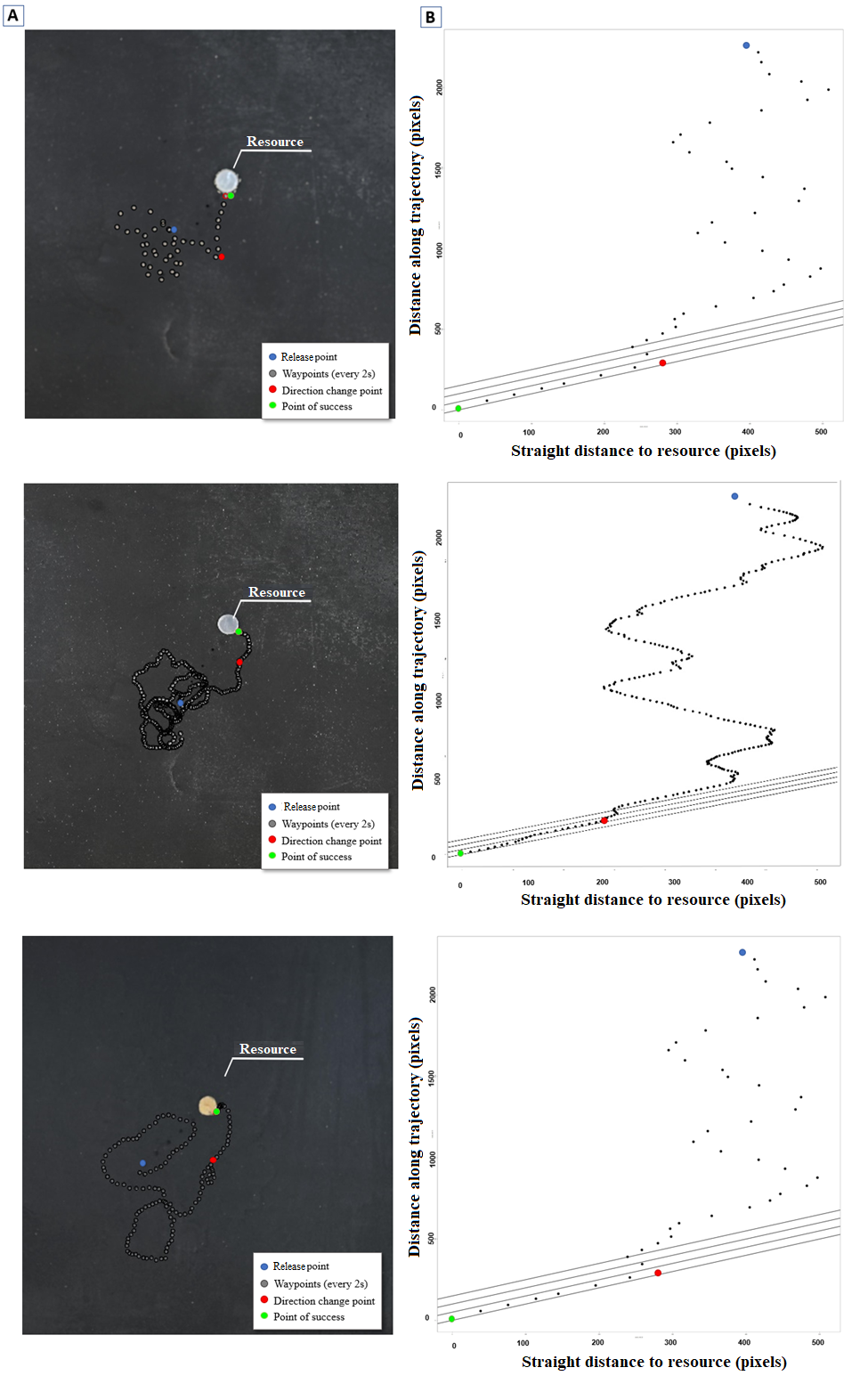


**Supplementary Fig. S3.** Example of prospected area calculation. The image is divided into grid-cell of 150 X 150 photo-pixels. We estimated the total prospected surface as the number of grid-cells in which the position points (x, y) density (light-green points) was higher than zero (grid-cell visited at least once). Grid-cell colours represent the density of points, from grey/brown (low) to dark-green (high).


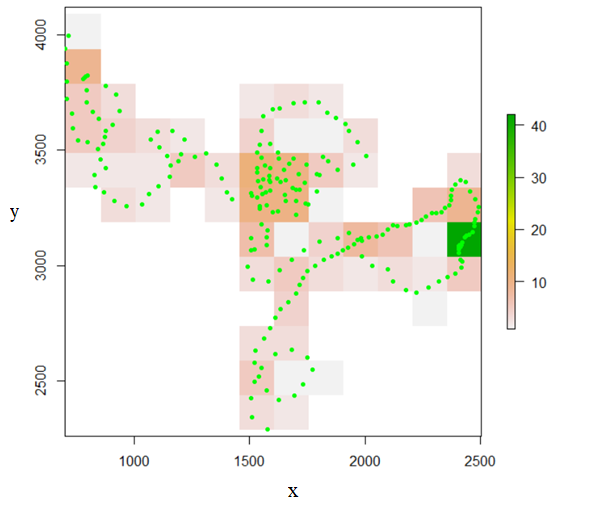


**Supplementary Table S1.** Multiple comparison test results using the Holm-Bonferroni method, after fitted a GLM with Quasi-poisson error distribution, log link function, final population size as the response variable and initial population size and resource quality as the explanatory variables (factors). Results are presented on the log scale. Significance level P-value < 0.05.

|  | **Contrast** | **Initial Population Size** | **Estimate** | **SE** | **Z. ratio** | **P-value** | **Group** |
| --- | --- | --- | --- | --- | --- | --- | --- |
|  | *(Agar-agar) - P.avium* | 10 | -0.16318 | 0.176614 | -0.92393 | 1 | a |
|  | *(Agar-agar) - A.opalus* | 10 | -0.16276 | 0.176631 | -0.92144 | 1 | a |
|  | *(Agar-agar) - Q.pubescens* | 10 | -0.14461 | 0.177372 | -0.81529 | 1 | a |
|  | *P.avium - A.opalus* | 10 | 0.000423 | 0.16929 | 0.002499 | 1 | a |
|  | *A.opalus - Q.pubescens* | 10 | 0.018145 | 0.170082 | 0.106687 | 1 | a |
|  | *P.avium - Q.pubescens* | 10 | 0.018568 | 0.170064 | 0.109185 | 1 | a |
|  | *(Agar-agar) - P.avium* | 20 | -0.01696 | 0.135058 | -0.12558 | 1 | a |
|  | *(Agar-agar) - A.opalus* | 20 | 0.009 | 0.135936 | 0.066211 | 1 | a |
|  | *P.avium - A.opalus* | 20 | 0.025961 | 0.135366 | 0.191787 | 1 | a |
|  | *A.opalus - Q.pubescens* | 20 | 0.41096 | 0.152574 | 2.693504 | **0.0291** | a |
|  | *(Agar-agar) - Q.pubescens* | 20 | 0.41996 | 0.152301 | 2.757426 | **0.0291** | a |
|  | *P.avium - Q.pubescens* | 20 | 0.436921 | 0.151793 | 2.878401 | **0.0240** | a |
|  | *P.avium - A.opalus* | 40 | -0.30027 | 0.126085 | -2.38152 | **0.0345** | a |
|  | *(Agar-agar) - A.opalus* | 40 | 0.030578 | 0.115433 | 0.264899 | 0.7911 | b |
|  | *(Agar-agar) - P.avium* | 40 | 0.330852 | 0.125275 | 2.641013 | **0.0248** | bc |
|  | *P.avium - Q.pubescens* | 40 | 0.834218 | 0.173687 | 4.802989 | **<0.0001** | cd |
|  | *A.opalus - Q.pubescens* | 40 | 1.134492 | 0.166728 | 6.804451 | **<0.0001** | de |
|  | *(Agar-agar) - Q.pubescens* | 40 | 1.16507 | 0.166116 | 7.0136 | **<0.0001** | e |
|  | *P.avium - A.opalus* | 60 | -0.16061 | 0.10628 | -1.51122 | 0.2615 | a |
|  | *(Agar-agar) - A.opalus* | 60 | 0.137215 | 0.098612 | 1.391467 | 0.2615 | b |
|  | *(Agar-agar) - P.avium* | 60 | 0.297828 | 0.103099 | 2.888749 | **0.0116** | ab |
|  | *P.avium - Q.pubescens* | 60 | 0.999185 | 0.150563 | 6.636318 | **<0.0001** | c |
|  | *A.opalus - Q.pubescens* | 60 | 1.159798 | 0.147527 | 7.861618 | **<0.0001** | cd |
|  | *(Agar-agar) - Q.pubescens* | 60 | 1.297013 | 0.145251 | 8.929428 | **<0.0001** | d |
|  | *P.avium - A.opalus* | 80 | -0.23937 | 0.094383 | -2.53621 | **0.022** | a |
|  | *(Agar-agar) - A.opalus* | 80 | 0.013517 | 0.088285 | 0.153109 | 0.878 | b |
|  | *(Agar-agar) - P.avium* | 80 | 0.252891 | 0.094103 | 2.687382 | **0.022** | c |
|  | *P.avium - Q.pubescens* | 80 | 1.001645 | 0.136222 | 7.353043 | **<0.0001** | d |
|  | *A.opalus - Q.pubescens* | 80 | 1.241018 | 0.13227 | 9.382487 | **<0.0001** | e |
|  | *(Agar-agar) - Q.pubescens* | 80 | 1.254536 | 0.13207 | 9.498993 | **<0.0001** | e |

**Supplementary Table S2.** Multiple comparison test results using the Holm-Bonferroni method, after fitted a GLM with Negative-binomial error distribution, log link function, reproduction rate as the response variable and initial population size and resource quality as the explanatory variables (factors). Results are presented on the log scale. Significance level P < 0.05.

|  | **Contrast** | **Initial Population Size** | **Estimate** | **SE** | **Z-ratio** | **P-value** | **Group** |
| --- | --- | --- | --- | --- | --- | --- | --- |
|  | *(Agar-agar) - P.avium* | 10 | -0.16318 | 0.11871 | -1.37458 | 1 | a |
|  | *(Agar-agar) - A.opalus* | 10 | -0.16276 | 0.11872 | -1.37097 | 1 | a |
|  | *(Agar-agar) - Q.pubescens* | 10 | -0.14461 | 0.11888 | -1.21645 | 1 | a |
|  | *P.avium - A.opalus* | 10 | 0.00042 | 0.11713 | 0.00361 | 1 | a |
|  | *A.opalus - Q.pubescens* | 10 | 0.01815 | 0.11730 | 0.15470 | 1 | a |
|  | *P.avium - Q.pubescens* | 10 | 0.01857 | 0.11729 | 0.15831 | 1 | a |
|  | *(Agar-agar) - P.avium* | 20 | -0.01695 | 0.12194 | -0.13898 | 1 | ab |
|  | *(Agar-agar) - A.opalus* | 20 | 0.00899 | 0.12222 | 0.07358 | 1 | ab |
|  | *P.avium - A.opalus* | 20 | 0.02594 | 0.12204 | 0.21256 | 1 | ac |
|  | ***A.opalus - Q.pubescens*** | **20** | **0.41096** | **0.12789** | **3.21346** | **0.0052** | **abcd** |
|  | ***(Agar-agar) - Q.pubescens*** | **20** | **0.41995** | **0.12779** | **3.28624** | **0.0051** | **cd** |
|  | ***P.avium - Q.pubescens*** | **20** | **0.43690** | **0.12761** | **3.42364** | **0.0037** | **bd** |
|  | *P.avium - A.opalus* | 40 | -0.29933 | 0.13735 | -2.17927 | 0.0586 | a |
|  | *(Agar-agar) - A.opalus* | 40 | 0.03028 | 0.13173 | 0.22983 | 0.8182 | a |
|  | ***(Agar-agar) - P.avium*** | **40** | **0.32960** | **0.13692** | **2.40730** | **0.0482** | **ab** |
|  | ***P.avium - Q.pubescens*** | **40** | **0.83339** | **0.16512** | **5.04716** | **<0.0001** | **bc** |
|  | ***A.opalus - Q.pubescens*** | **40** | **1.13272** | **0.16085** | **7.04219** | **<0.0001** | **c** |
|  | ***(Agar-agar) - Q.pubescens*** | **40** | **1.16300** | **0.16048** | **7.24710** | **<0.0001** | **c** |
|  | *P.avium - A.opalus* | 60 | -0.16030 | 0.13960 | -1.14831 | 0.5017 | a |
|  | *(Agar-agar) - A.opalus* | 60 | 0.13732 | 0.13453 | 1.02074 | 0.5017 | a |
|  | *(Agar-agar) - P.avium* | 60 | 0.29762 | 0.13747 | 2.16502 | 0.0912 | ab |
|  | ***P.avium - Q.pubescens*** | **60** | **0.99899** | **0.17190** | **5.81136** | **<0.0001** | **bc** |
|  | ***A.opalus - Q.pubescens*** | **60** | **1.15929** | **0.16956** | **6.83703** | **<0.0001** | **c** |
|  | ***(Agar-agar) - Q.pubescens*** | **60** | **1.29661** | **0.16781** | **7.72659** | **<0.0001** | **c** |
|  | *P.avium - A.opalus* | 80 | -0.24019 | 0.14145 | -1.69803 | 0.2147 | a |
|  | *(Agar-agar) - A.opalus* | 80 | 0.01426 | 0.13670 | 0.10429 | 0.9169 | a |
|  | *(Agar-agar) - P.avium* | 80 | 0.25445 | 0.14122 | 1.80176 | 0.2147 | a |
|  | ***P.avium - Q.pubescens*** | **80** | **0.99786** | **0.17707** | **5.63552** | **<0.0001** | **b** |
|  | ***A.opalus - Q.pubescens*** | **80** | **1.23805** | **0.17348** | **7.13664** | **<0.0001** | **b** |
|  | ***(Agar-agar) - Q.pubescens*** | **80** | **1.25231** | **0.17329** | **7.22669** | **<0.0001** | **b** |

**Supplementary Table S3.** Time-to-event analysis results of probability success in foraging using Kaplan-Meier estimator by release distance. The table summarizes the median and 95% confidence interval.

|  | ***strata*** | ***time*** | ***n.risk*** | ***n.event*** | ***surv*** | ***std.err*** | ***lower*** | ***upper*** |
| --- | --- | --- | --- | --- | --- | --- | --- | --- |
| 1 | distance=1 | 0.2 | 28 | 2 | 0.929 | 0.049 | 0.743 | 0.982 |
| 2 | distance=1 | 0.4 | 26 | 1 | 0.893 | 0.058 | 0.704 | 0.964 |
| 3 | distance=1 | 0.6 | 25 | 1 | 0.857 | 0.066 | 0.663 | 0.944 |
| 4 | distance=1 | 0.8 | 24 | 4 | 0.714 | 0.085 | 0.509 | 0.846 |
| 5 | distance=1 | 1 | 20 | 1 | 0.679 | 0.088 | 0.473 | 0.818 |
| 6 | distance=1 | 1.2 | 19 | 2 | 0.607 | 0.092 | 0.404 | 0.760 |
| 7 | distance=1 | 1.4 | 17 | 3 | 0.500 | 0.094 | 0.306 | 0.666 |
| 8 | distance=1 | 1.8 | 14 | 2 | 0.429 | 0.094 | 0.246 | 0.600 |
| 9 | distance=1 | 2.2 | 12 | 1 | 0.393 | 0.092 | 0.217 | 0.565 |
| 10 | distance=1 | 3 | 11 | 1 | 0.357 | 0.091 | 0.189 | 0.530 |
| 11 | distance=1 | 4 | 10 | 1 | 0.321 | 0.088 | 0.161 | 0.493 |
| 12 | distance=1 | 4.2 | 9 | 1 | 0.286 | 0.085 | 0.135 | 0.456 |
| 13 | distance=1 | 6.4 | 8 | 1 | 0.250 | 0.082 | 0.111 | 0.418 |
| 14 | distance=1 | 6.8 | 7 | 1 | 0.214 | 0.078 | 0.087 | 0.378 |
| 15 | distance=1 | 7.6 | 6 | 1 | 0.179 | 0.072 | 0.065 | 0.337 |
| 16 | distance=1 | 10.4 | 5 | 1 | 0.143 | 0.066 | 0.045 | 0.295 |
| 17 | distance=1 | 17.4 | 4 | 1 | 0.107 | 0.058 | 0.027 | 0.251 |
| 18 | distance=1 | 18.8 | 3 | 1 | 0.071 | 0.049 | 0.013 | 0.204 |
| 19 | distance=1 | 25.8 | 2 | 1 | 0.036 | 0.035 | 0.003 | 0.154 |
| 20 | distance=1 | 56.4 | 1 | 1 | 0.000 | NA | NA | NA |
| 21 | distance=2 | 0.6 | 16 | 1 | 0.938 | 0.061 | 0.632 | 0.991 |
| 22 | distance=2 | 2.2 | 15 | 1 | 0.875 | 0.083 | 0.586 | 0.967 |
| 23 | distance=2 | 3.4 | 14 | 1 | 0.813 | 0.098 | 0.525 | 0.935 |
| 24 | distance=2 | 5.6 | 13 | 2 | 0.688 | 0.116 | 0.405 | 0.856 |
| 25 | distance=2 | 8.4 | 11 | 1 | 0.625 | 0.121 | 0.349 | 0.811 |
| 26 | distance=2 | 8.6 | 10 | 1 | 0.563 | 0.124 | 0.295 | 0.762 |
| 27 | distance=2 | 9.8 | 9 | 1 | 0.500 | 0.125 | 0.245 | 0.710 |
| 28 | distance=2 | 10.6 | 8 | 1 | 0.438 | 0.124 | 0.198 | 0.656 |
| 29 | distance=2 | 11.2 | 7 | 1 | 0.375 | 0.121 | 0.154 | 0.598 |
| 30 | distance=2 | 14.4 | 6 | 1 | 0.313 | 0.116 | 0.114 | 0.536 |
| 31 | distance=2 | 20.2 | 5 | 1 | 0.250 | 0.108 | 0.078 | 0.472 |
| 32 | distance=2 | 24.8 | 4 | 1 | 0.188 | 0.098 | 0.046 | 0.402 |
| 33 | distance=2 | 26.2 | 3 | 1 | 0.125 | 0.083 | 0.021 | 0.328 |
| 34 | distance=2 | 27.8 | 2 | 1 | 0.063 | 0.061 | 0.004 | 0.247 |
| 35 | distance=2 | 31.8 | 1 | 1 | 0.000 | NA | NA | NA |
| 36 | distance=3 | 0.8 | 14 | 1 | 0.929 | 0.069 | 0.591 | 0.990 |
| 37 | distance=3 | 2.8 | 13 | 1 | 0.857 | 0.094 | 0.539 | 0.962 |
| 38 | distance=3 | 4 | 12 | 2 | 0.714 | 0.121 | 0.406 | 0.882 |
| 39 | distance=3 | 7 | 10 | 1 | 0.643 | 0.128 | 0.343 | 0.833 |
| 40 | distance=3 | 7.4 | 9 | 1 | 0.571 | 0.132 | 0.284 | 0.780 |
| 41 | distance=3 | 9 | 8 | 1 | 0.500 | 0.134 | 0.229 | 0.722 |
| 42 | distance=3 | 9.8 | 7 | 1 | 0.429 | 0.132 | 0.177 | 0.660 |
| 43 | distance=3 | 15.8 | 6 | 1 | 0.357 | 0.128 | 0.130 | 0.594 |
| 44 | distance=3 | 19.8 | 5 | 1 | 0.286 | 0.121 | 0.088 | 0.524 |
| 45 | distance=3 | 23.8 | 4 | 1 | 0.214 | 0.110 | 0.052 | 0.448 |
| 46 | distance=3 | 44.6 | 3 | 1 | 0.143 | 0.094 | 0.023 | 0.366 |
| 47 | distance=3 | 45 | 2 | 1 | 0.071 | 0.069 | 0.005 | 0.275 |
| 48 | distance=3 | 48.2 | 1 | 1 | 0.000 | NA | NA | NA |
| 49 | distance=4 | 4.2 | 13 | 1 | 0.923 | 0.074 | 0.566 | 0.989 |
| 50 | distance=4 | 7.2 | 12 | 1 | 0.846 | 0.100 | 0.512 | 0.959 |
| 51 | distance=4 | 8.4 | 11 | 1 | 0.769 | 0.117 | 0.442 | 0.919 |
| 52 | distance=4 | 8.8 | 10 | 2 | 0.615 | 0.135 | 0.308 | 0.818 |
| 53 | distance=4 | 13.2 | 8 | 1 | 0.538 | 0.138 | 0.248 | 0.760 |
| 54 | distance=4 | 23.8 | 7 | 1 | 0.462 | 0.138 | 0.192 | 0.696 |
| 55 | distance=4 | 24 | 6 | 1 | 0.385 | 0.135 | 0.141 | 0.628 |
| 56 | distance=4 | 25.4 | 5 | 1 | 0.308 | 0.128 | 0.095 | 0.554 |
| 57 | distance=4 | 31 | 4 | 1 | 0.231 | 0.117 | 0.056 | 0.475 |
| 58 | distance=4 | 32.2 | 3 | 1 | 0.154 | 0.100 | 0.025 | 0.388 |
| 59 | distance=4 | 41.4 | 2 | 1 | 0.077 | 0.074 | 0.005 | 0.292 |
| 60 | distance=4 | 53.6 | 1 | 1 | 0.000 | NA | NA | NA |
| 61 | distance=5 | 3.8 | 9 | 1 | 0.889 | 0.105 | 0.433 | 0.984 |
| 62 | distance=5 | 7.8 | 8 | 1 | 0.778 | 0.139 | 0.365 | 0.939 |
| 63 | distance=5 | 11.6 | 7 | 1 | 0.667 | 0.157 | 0.282 | 0.878 |
| 64 | distance=5 | 15.6 | 6 | 1 | 0.556 | 0.166 | 0.204 | 0.805 |
| 65 | distance=5 | 39.2 | 5 | 1 | 0.444 | 0.166 | 0.136 | 0.719 |
| 66 | distance=5 | 39.8 | 4 | 1 | 0.333 | 0.157 | 0.078 | 0.623 |
| 67 | distance=5 | 49 | 3 | 1 | 0.222 | 0.139 | 0.034 | 0.513 |
| 68 | distance=5 | 54.4 | 2 | 1 | 0.111 | 0.105 | 0.006 | 0.388 |
| 69 | distance=5 | 59 | 1 | 1 | 0.000 | NA | NA | NA |
